# Supplementary figures and images for: Early IL-17A production helps establish Mycobacterium intracellulare infection in mice
Source: PLoS Pathog. 2022 Apr 1;18(4):e1010454. doi: 10.1371/journal.ppat.1010454 (PMC9007361; doi:10.1371/journal.ppat.1010454)

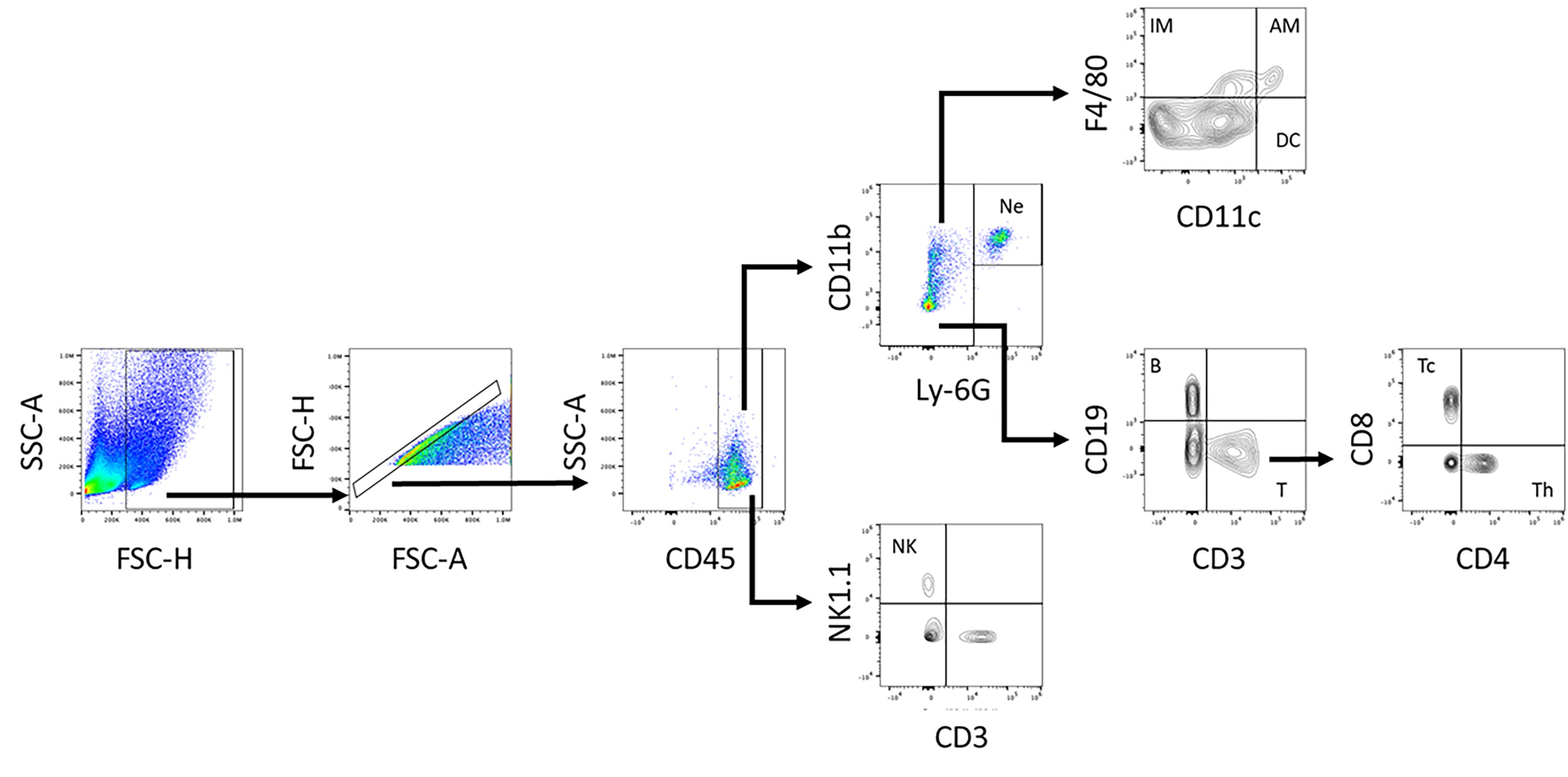

Supplement: S1 Fig — Mouse lung cells were stained with various antibodies (S1 Table), and immune cells were determined using the illustrated gating strategy. Neutrophils (Ne) were gated as CD45+CD11b+Ly6G+ cells. Interstitial macrophages (IMs) were gated as CD45+Ly6G-F4/80+CD11c-cells. Alveolar macrophages (AMs) were gated as CD45+Ly6G-F4/80+CD11c+ cells. Dendritic cells (DCs) were gated as CD45+Ly6G-F4/80-CD11c+ cells. B cells were gated as CD45+Ly6G-CD19+CD3- cells. T cells were gated as CD45+Ly6G-CD19-CD3+ cells. CD8 T (Tc) cells were gated as CD45+Ly6G-CD19-CD3+CD8+CD4- cells. CD4 T (Th) cells were gated as CD45+Ly6G-CD19-CD3+CD8-CD4+ cells. NK cells were gated as CD45+CD3-NK1.1+ cells. (TIF) [file ppat.1010454.s001.tif]

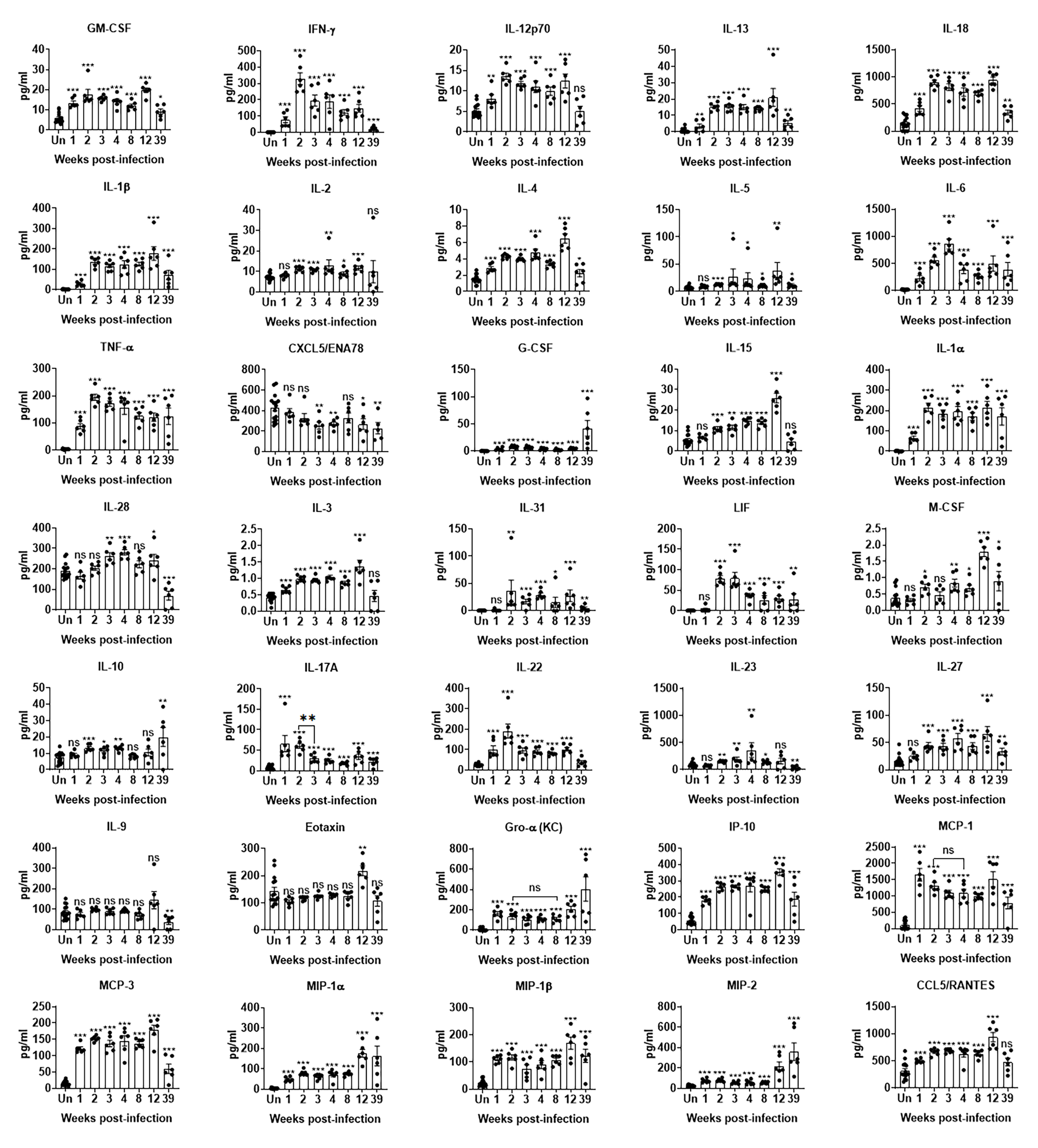

Supplement: S2 Fig — C57BL/6 mice were infected with clinical isolates of M. intracellulare (5 × 10^7^ CFU) via the intranasal route. Mice were sacrificed at 1, 2, 3, 4, 8, 12 and 39 weeks postinfection. Lungs were collected from uninfected control and M. intracellulare-infected mice, and 36 cytokine and chemokine levels were determined in lung homogenates of mice using multiplex ELISAs. IFN-α was under the limit of detection. Data were pooled from two independent experiments (uninfected mice n = 15, infected mice n = 6 mice per indicated time point). Data are expressed as the means ± SEM. *P < 0.05, **P < 0.01, and ***P < 0.001. ns, not significant. P values for comparison between uninfected mice (Un) and other were indicated with asterisks sans connecting lines. For other comparisons, connecting lines used. (TIF) [file ppat.1010454.s002.tif]

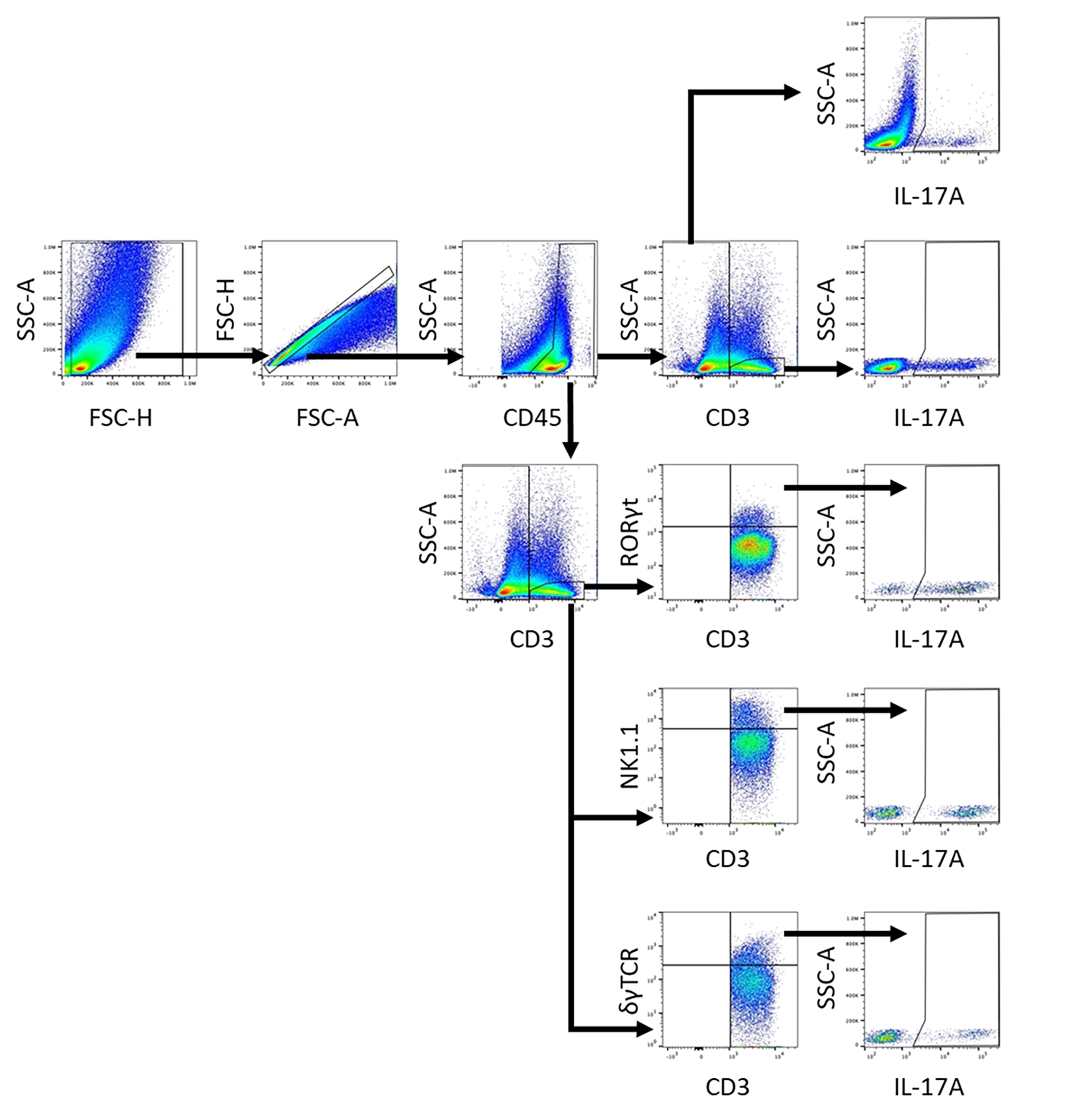

Supplement: S3 Fig — Mouse lung cells were stained with various antibodies (S1 Table), and the percentages (%) of IL-17A-producing cells in CD45+CD3- cells, CD45+CD3+ cells, CD45+CD3+RORγt+ cells, CD45+CD3+NK1.1+ cells and CD45+CD3+γδTCR+ cells were determined using the illustrated gating strategy. (TIF) [file ppat.1010454.s003.tif]

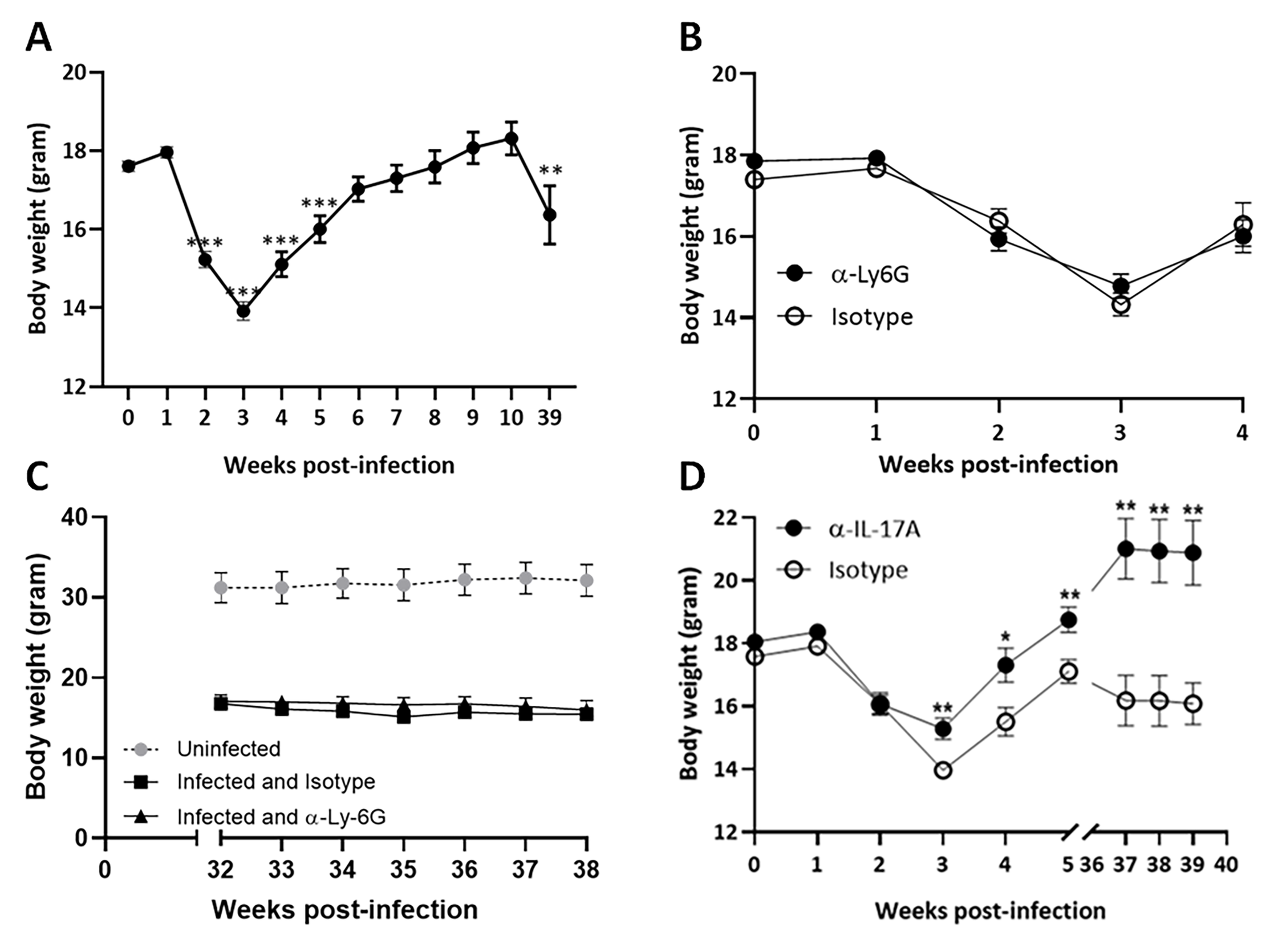

Supplement: S4 Fig — (A-D) C57BL/6 mice were infected with clinical isolates of M. intracellulare (5 × 10^7^ CFU) via the intranasal route. Body weight was monitored in M. intracellulare-infected mice (A), M. intracellulare-infected mice treated with anti-Ly6G mAb or isotype-matched control Ab in early infection (B), M. intracellulare-infected mice treated with anti-Ly6G mAb or isotype-matched control Ab in chronic infection (C) and M. intracellulare-infected mice treated with anti-IL-17A mAb or isotype-matched control Ab (D). Data are expressed as the means ± SEM. *P < 0.05, **P < 0.01, and ***P < 0.001 compared with initial body weight (A) or between anti-IL-17A mAb- or isotype-matched control Ab-treated mice (D). (TIF) [file ppat.1010454.s004.tif]

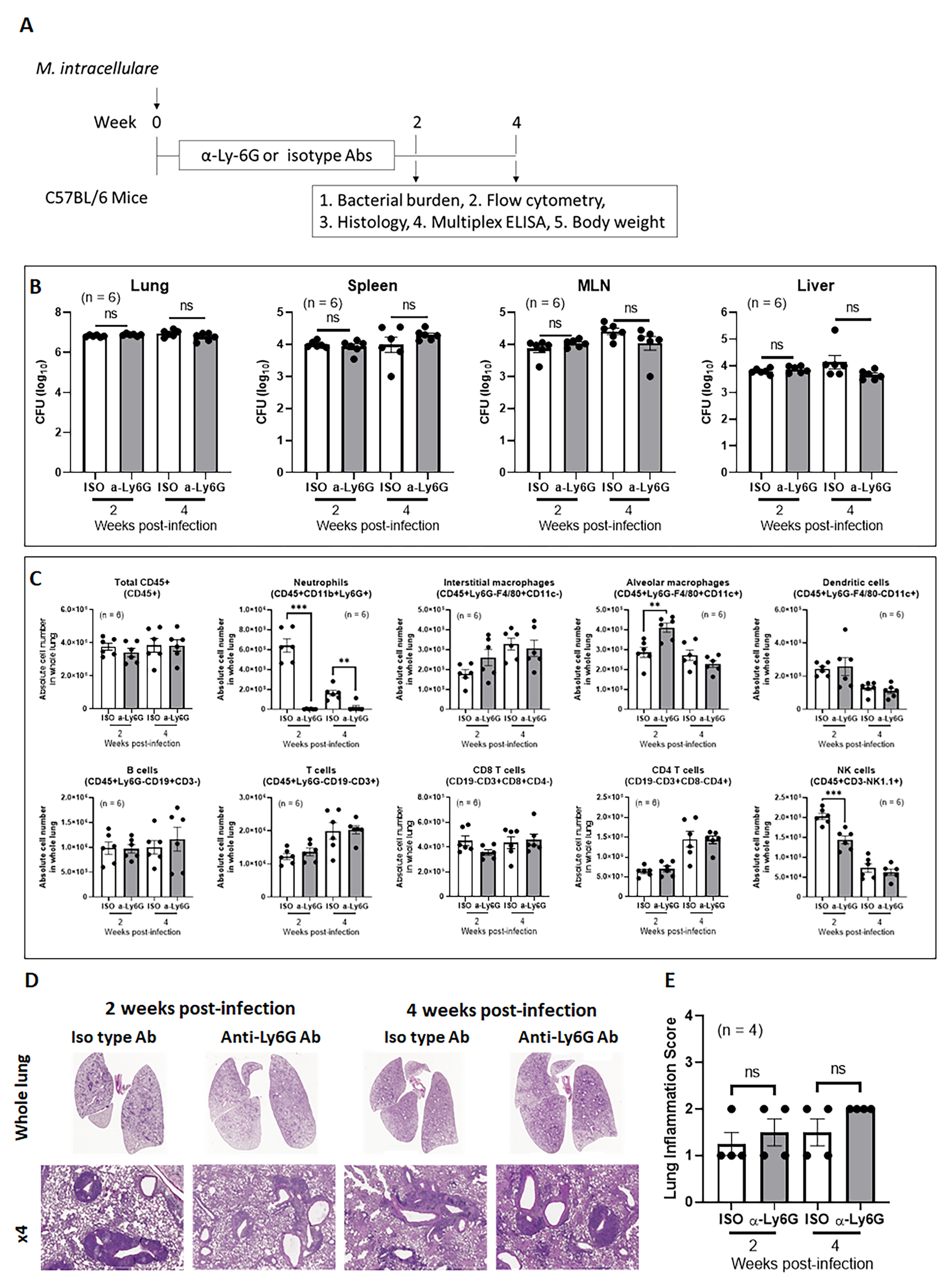

Supplement: S5 Fig — (A) Schematic representation of M. intracellulare infection and the experimental schedule for early neutrophil depletion are shown. C57BL/6 mice were infected with clinical isolates of M. intracellulare (5 × 10^7^ CFU) via the intranasal route. To deplete neutrophils, M. intracellulare-infected mice were intraperitoneally injected with anti-Ly6G mAb or rat IgG2a isotype control Ab every other day from 1 to 11 days postinfection. Lungs were collected at 2 and 4 weeks postinfection. (B) Bacterial burden in the lung, spleen, mediastinal lymph node (MLN) and liver was determined. (C) The absolute number of immune cells per whole lung was determined by flow cytometry. (D) Representative figures of lung histology are shown. (E) The severity of lung inflammation was quantified from a total of 4 mice per indicated time point using a score from 0 (no inflammation) to 4 (severe inflammation) for each of the following criteria: alveolar wall inflammation, alveolar destruction, leukocyte infiltration, and perivascular inflammation. Data were pooled from two independent experiments. Data are expressed as the means ± SEM. *P < 0.05, **P < 0.01, and ***P < 0.001. (TIF) [file ppat.1010454.s005.tif]

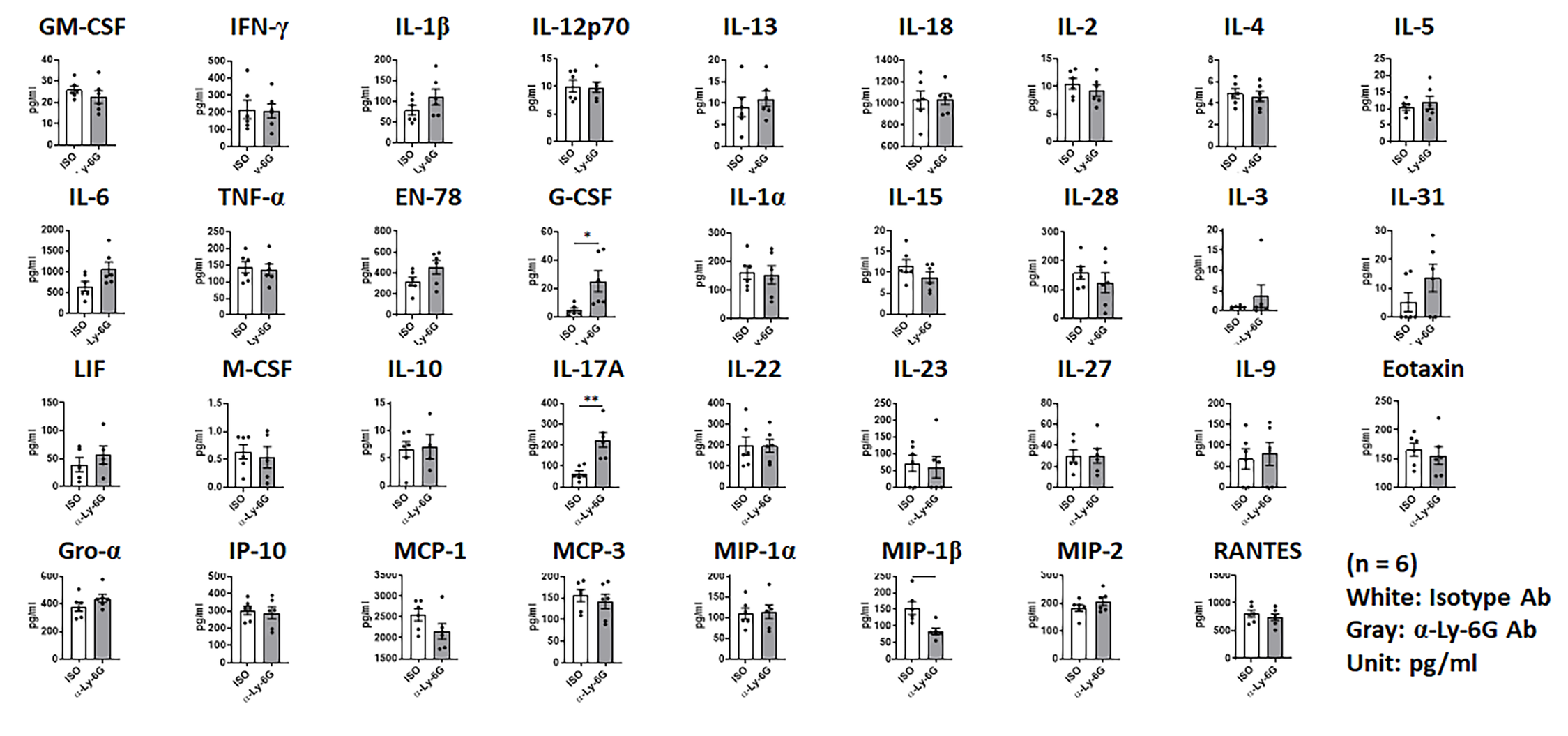

Supplement: S6 Fig — Cytokine and chemokine levels were determined in lung homogenates of mice at 2 weeks postinfection using multiplex ELISA. IFN-α was under limit of detection. Data were pooled from two independent experiments. Data are expressed as the means ± SEM. *P < 0.05, and **P < 0.01. (TIF) [file ppat.1010454.s006.tif]

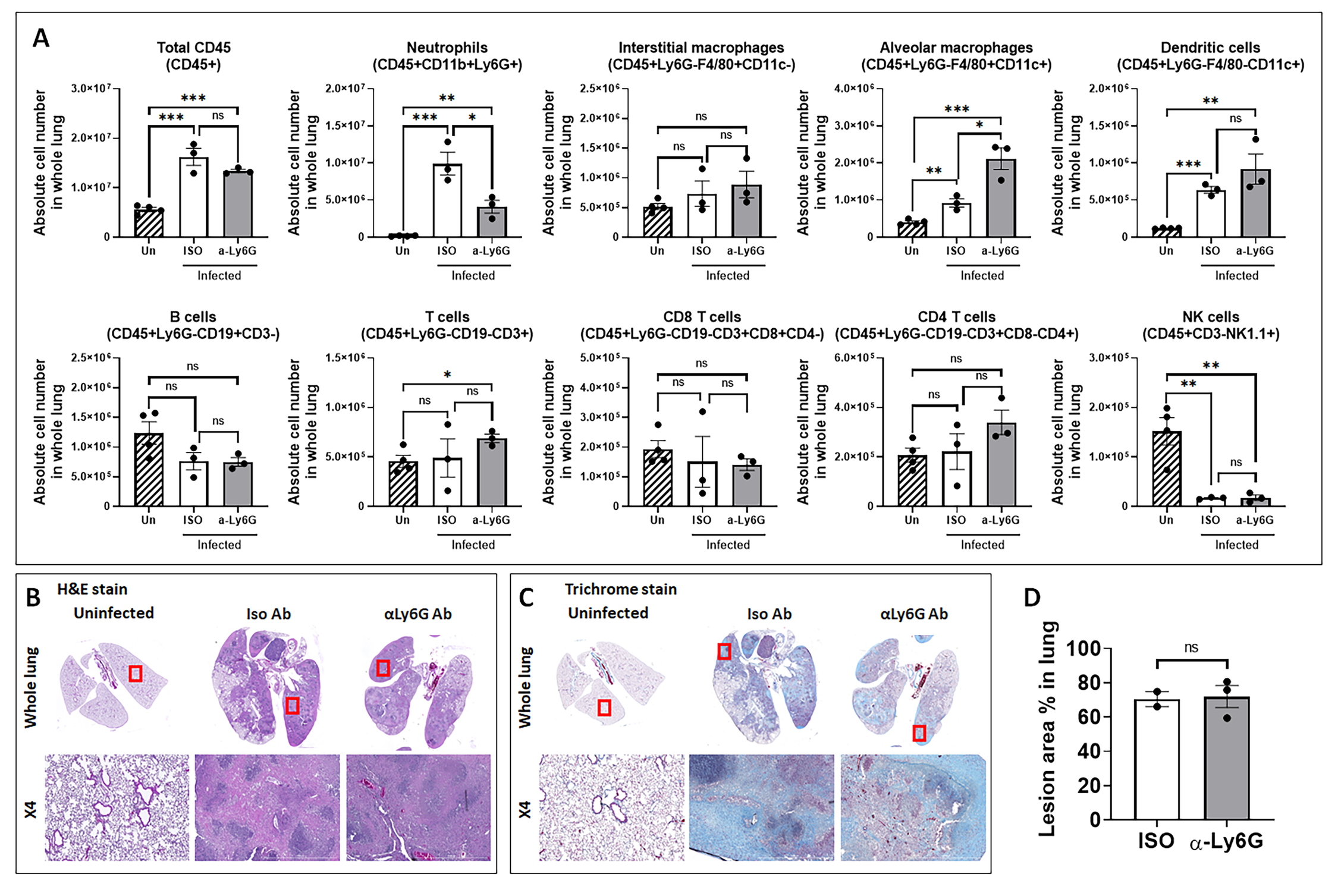

Supplement: S7 Fig — To deplete neutrophils during chronic infection, mice infected with M. intracellulare were intraperitoneally injected with an initial dose of 400 μg followed by 300 μg of anti-Ly6G antibody (clone 1A8, BioXcell) or an initial dose of 400 μg followed by 300 μg of rat IgG2a isotype control (clone 2A3, BioXcell) 3 times per week for 2 weeks from 32 to 34 weeks postinfection. (A) The absolute number of immune cells per whole lung was determined at 38 weeks postinfection by flow cytometry. (B-D) Representative images of H&E staining (B) and trichrome staining (C) of each group are shown. The lesion area (%) in lung images (D) was determined based on whole lung images stained with H&E at 38 weeks postinfection. Data were pooled from two independent experiments. Data are expressed as the means ± SEM. *P < 0.05, **P < 0.01, and ***P < 0.001. ns, not significant. (TIF) [file ppat.1010454.s007.tif]

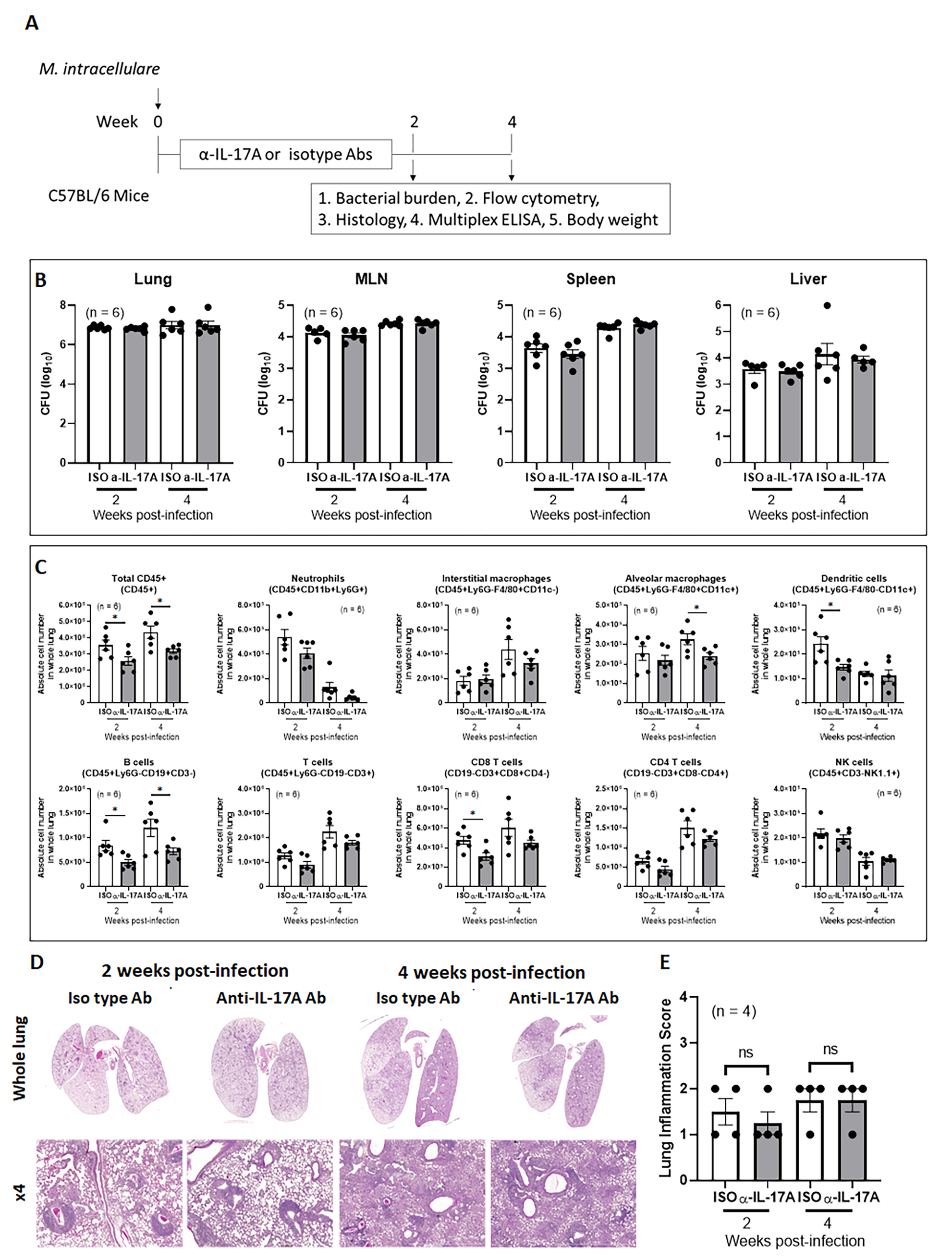

Supplement: S8 Fig — (A) Schematic representation of M. intracellulare infection and the experimental schedule for neutralization of IL-17A during early infection are shown. C57BL/6 mice were infected with clinical isolates of M. intracellulare (5 × 10^7^ CFU) via intranasal delivery. To neutralize IL-17A, M. intracellulare-infected mice were intraperitoneally injected with anti-IL-17A mAb or IgG1 isotype control Ab every other day from 1 to 11 days postinfection. Lungs were collected at 2 and 4 weeks postinfection. (B) Bacterial burden in the lung, spleen, mediastinal lymph node (MLN) and liver was determined. (C) The absolute number of immune cells per whole lung was determined by flow cytometry. (D) Representative figures of lung histology are shown. (E) The severity of lung inflammation was quantified in 4 mice at each indicated time point using a score from 0 (no inflammation) to 4 (severe inflammation) for each of the following criteria: alveolar wall inflammation, alveolar destruction, leukocyte infiltration, and perivascular inflammation. Data were pooled from two independent experiments. Data are expressed as the means ± SEM. *P < 0.05. ns, not significant. (TIF) [file ppat.1010454.s008.tif]

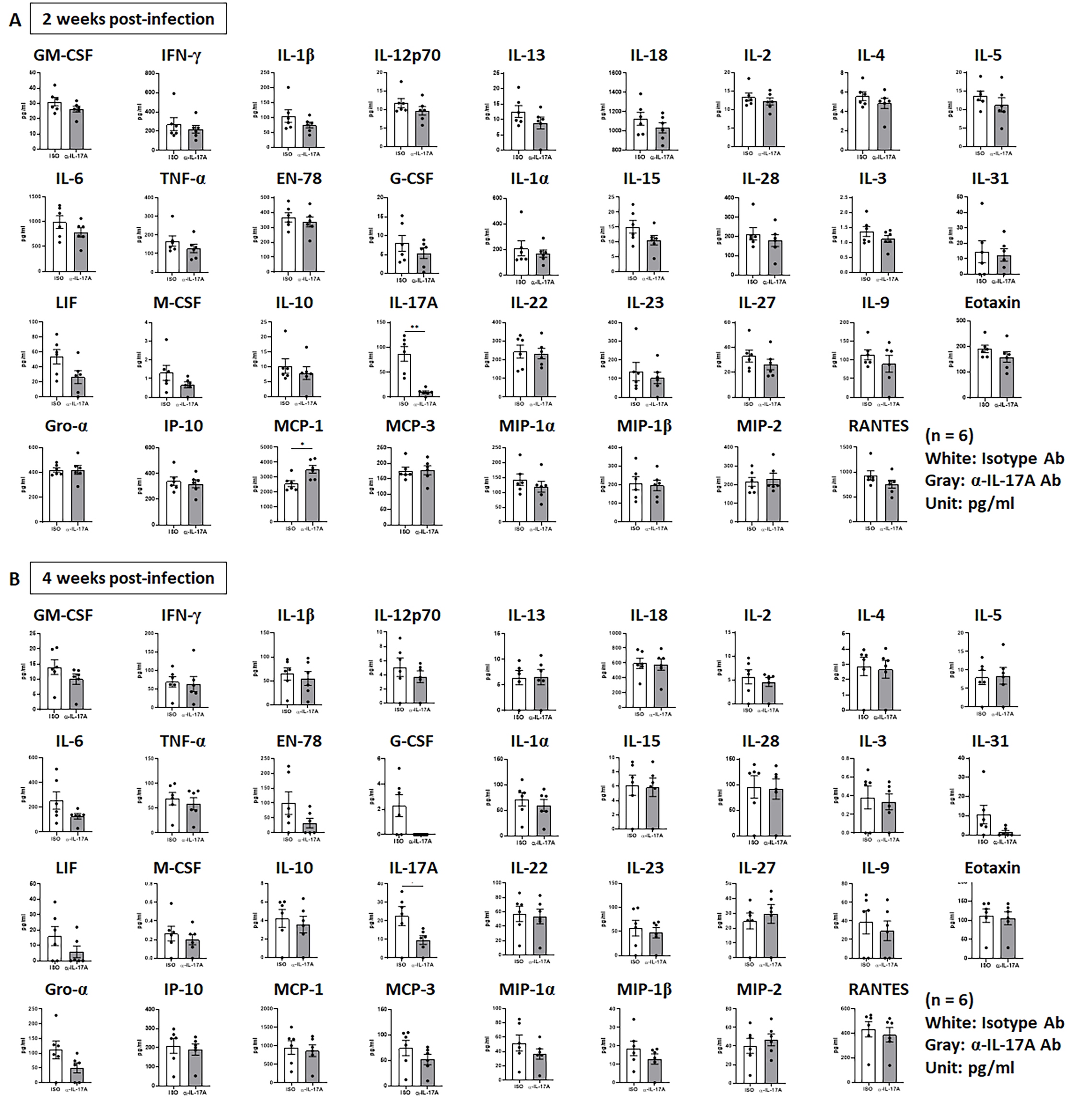

Supplement: S9 Fig — (A, B) Cytokine and chemokine levels were determined in lung homogenates of M. intracellulare-infected mice treated with anti-IL-17A mAb or isotype-matched control Ab at 2 weeks (A) and 4 weeks postinfection (B) using multiplex ELISA. IFN-α was under limit of detection. Data were pooled from two independent experiments. Data are expressed as the means ± SEM. *P < 0.05 and **P < 0.01. (TIF) [file ppat.1010454.s009.tif]

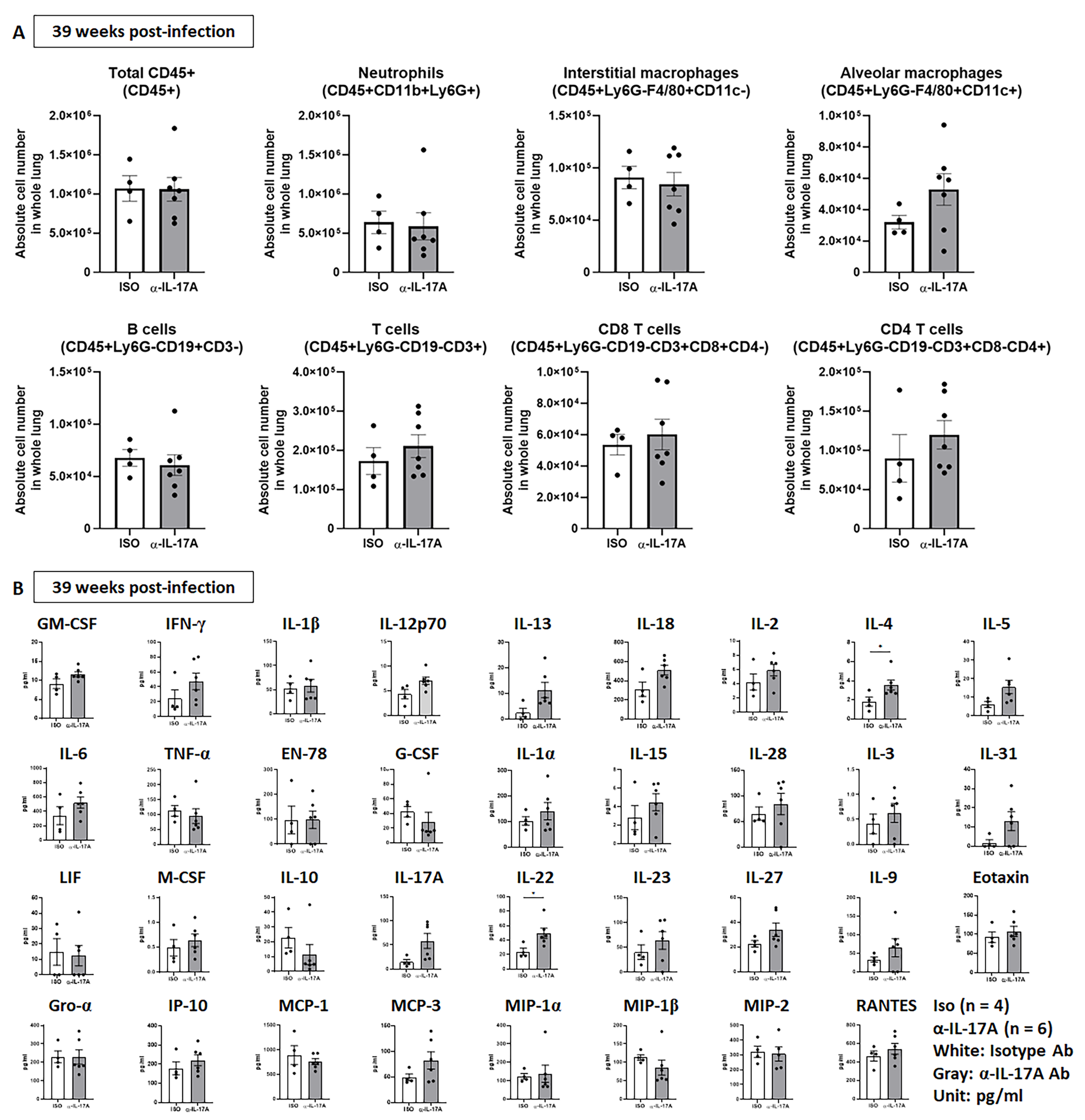

Supplement: S10 Fig — (A, B) The absolute number of immune cells per whole lung was determined in lung homogenates of M. intracellulare-infected mice treated with anti-IL-17A mAb or isotype-matched control at 39 weeks postinfection by flow cytometry (A). Cytokine and chemokine levels were determined in lung homogenates of mice at 39 weeks postinfection using multiplex ELISA (B). IFN-α was under limit of detection. Data were pooled from two independent experiments. Data are expressed as the means ± SEM. *P < 0.05. (TIF) [file ppat.1010454.s010.tif]

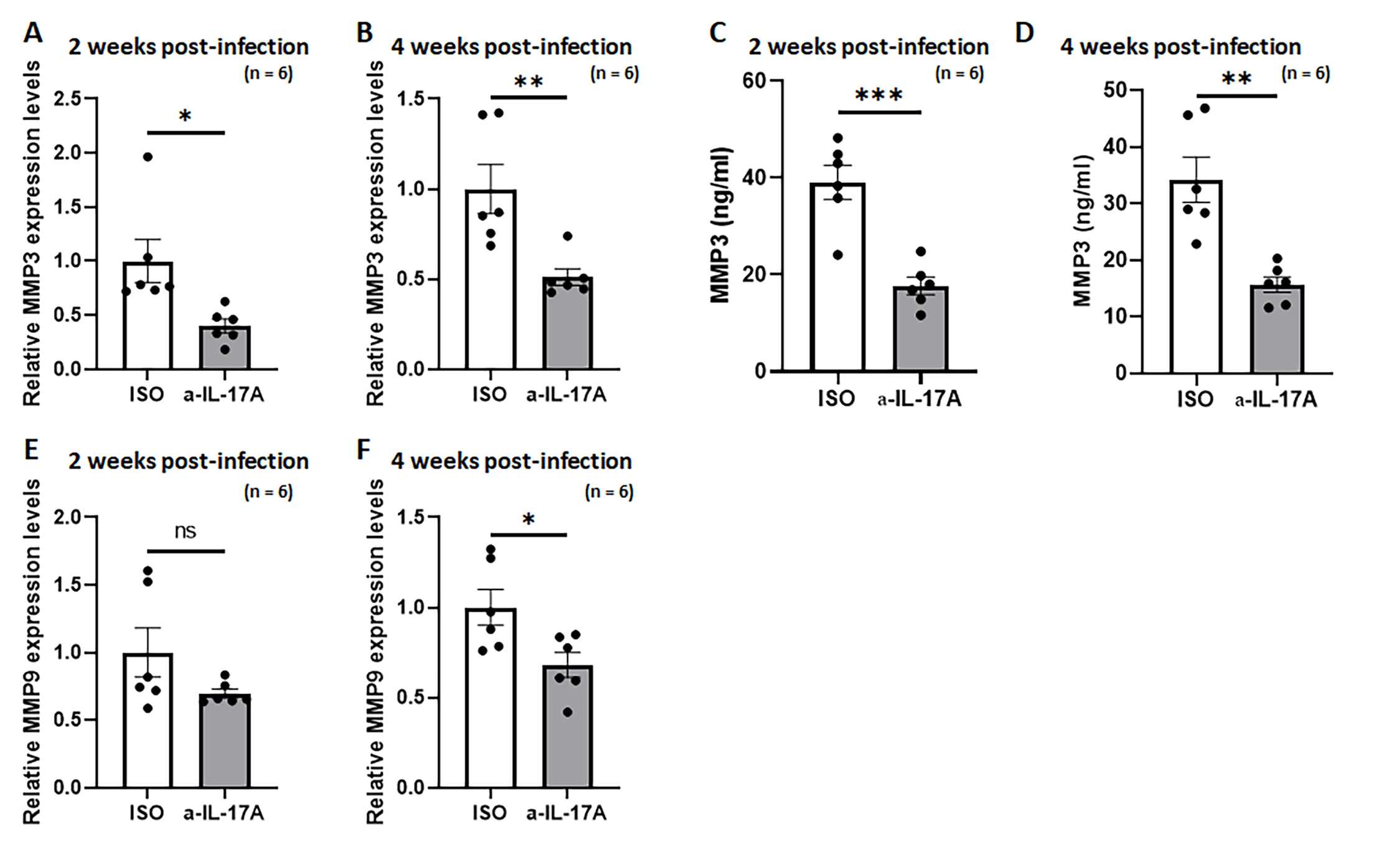

Supplement: S11 Fig — (A-F) RNA expression levels (A, B) and protein levels (C, D) of MMP-3 and RNA expression levels of MMP-9 (E, F) were determined from the lungs of M. intracellulare-infected mice treated with anti-IL-17A mAb or isotype-matched control at 2 and 4 weeks postinfection. Data were pooled from two independent experiments (total n = 6 mice per group at each indicated time point). Data are expressed as the means ± SEM. *P < 0.05, **P < 0.01, and ***P < 0.001. (TIF) [file ppat.1010454.s011.tif]

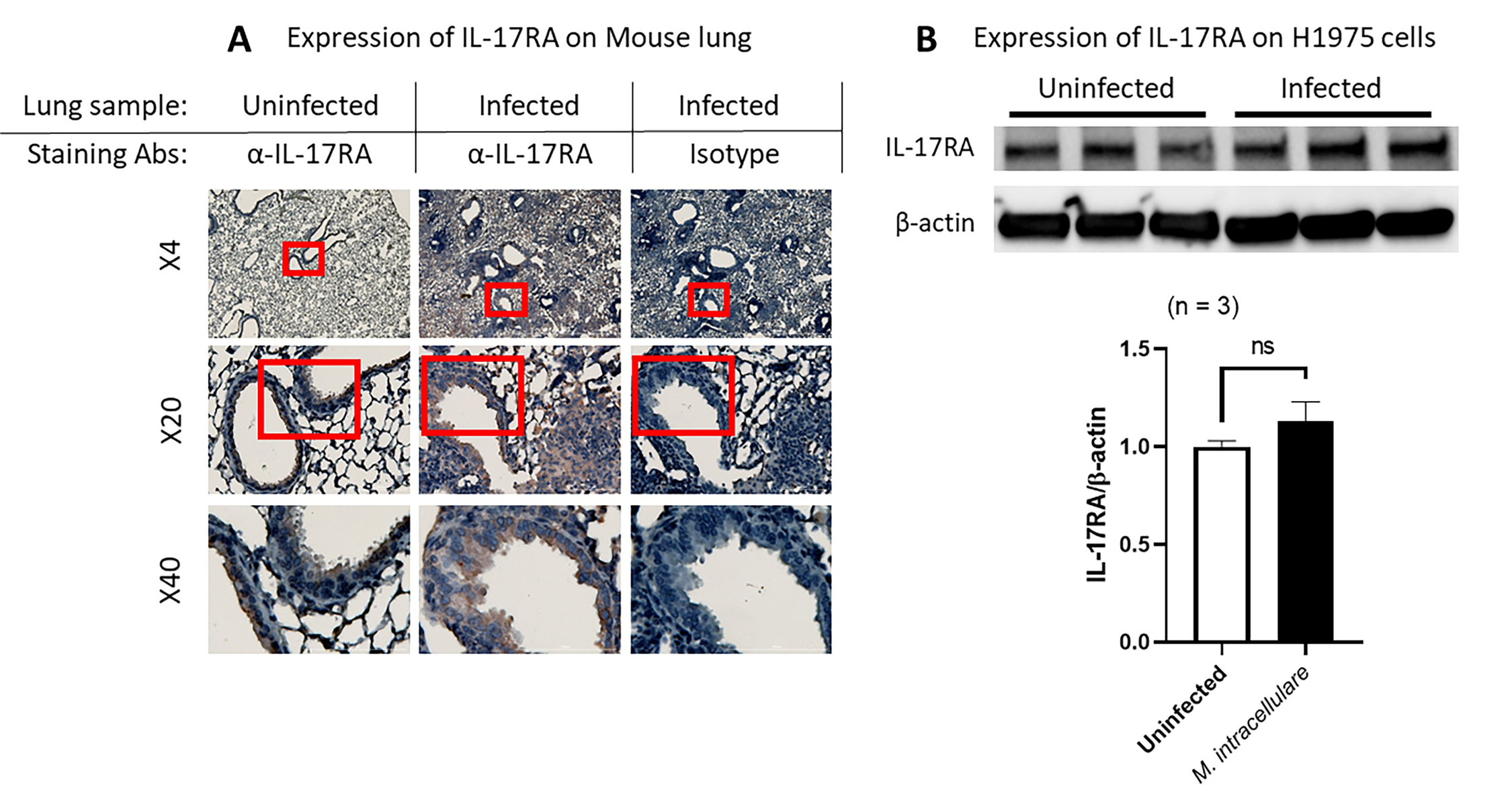

Supplement: S12 Fig — (A) Immunohistochemical staining was performed to determine the expression of IL-17RA in the lungs of uninfected mice or M. intracellulare-infected mice after 2 weeks of infection. (B) IL-17RA expression was determined from the lysate of H1975 cells after 1 day of infection by western blot. (C) The intensity of each target protein band was normalized to that of β-actin. Three independent experiments were performed. Data are expressed as the means ± SEM. (TIF) [file ppat.1010454.s012.tif]
