# Supplementary material for: Early IL-17A production helps establish Mycobacterium intracellulare infection in mice
Source: PLoS Pathog. 2022 Apr 1;18(4):e1010454. doi: 10.1371/journal.ppat.1010454 (PMC9007361; doi:10.1371/journal.ppat.1010454)
Supplement: Attachment — Submitted filename: Responses to the reviews (02102022).docx [file ppat.1010454.s014.docx]

Dear Editor,

We thank the reviewers for their suggestions and comments to strengthen the manuscript. We enclose a revised manuscript entitled, **“Early IL-17A production helps establish *Mycobacterium intracellulare* infection in mice”**, to be considered for publication in the *PLOS Pathogens*. We have addressed all of the reviewers’ unedited comments point-by-point as described below.

**Major Issues: Key Experiments Required for Acceptance**

**Reviewer #1:**

**Q1.** My primary experimental concern is with Figure 7; as the only in vitro data presented, I think it would benefit from a few additional conditions and/or readouts. For Fig 7A, it would be useful to see additional concentrations of IL-17A or different bacterial MOIs in order to see that more or less IL-17A/bacteria leads to higher or lower MMP-3 production. Looking at MMP-9 production in this set of experiments is also crucial, since it would strengthen the conclusion that MMP-3 is specifically regulated by IL-17A. In Fig 7B, it would be helpful to see CFUs at more than one timepoint to get a sense of the dynamics; are there differences in CFUs earlier during infection? Or later during infection? Are bacteria replicating in this cell line or being killed over time? Measuring cell death (LDH assay or something similar) might also strengthen this panel: if treatment leads to increased death or survival but there equal CFUs are recovered, there would be an overall difference in the number of bacteria per cell, which would be biologically meaningful.

**Response:** We now performed these experiments and results are shown in Fig. 7 and described on page 12 – 13, line 266 – 282.

**Q2.** My other major concern is understanding how the authors developed this infection model. The authors provide a nice description of similar mouse models and their shortcomings in the discussion, but were similar preliminary experiments done by the authors in the process of developing this model? Is there data that can be included from the authors trying other infection routes, other mouse strains, or different inoculums? Anything that would help establish how and why the authors chose these parameters for the rest of the paper. At the very least, a discussion of this decision-making process at the beginning of the results section would be beneficial.

**Response:** As described in discussion (page 14 – 15, line 295 – 321), a low dose (1 X 10^4^ CFU) – a medium dose (1 X 10^5^ CFU) of *M. avium* complex infection did not cause chronic infection in mice. In the current study we demonstrated that at high infection dose is required to establish *M. intracellulare* chronic infection. Our high dose model can therefore enable better understanding of host immune responses. Testing of alternative infection routes, other mouse strains, or different inoculums would require significant additional allocation of animals, which we attempted to mitigate.

**Q3.** I’m wondering why mice treated with anti-Ly6G weren’t fully analyzed (CFUs, histology, cytokines, cell analysis) at week 39 or 48. I’m especially curious about this since in mice treated with anti-IL-17A mAb, some key phenotypes like differences in CFUs and histology weren’t apparent until this late timepoint. If the late time point had been examined for the anti-Ly6G mice, would phenotypes be detectable?

**Response:** Anti-Ly6G Ab treatment during early infection (0 – 2 weeks of infection) had no effect on the mortality of the infected mice. We thought it is not necessary to perform additional experiments. We performed new experiments requested by reviewer #3. Mice were treated with anti-Ly6G Ab or isotype control Ab during 32–34 weeks of infection and CFUs, histology and immune cell subtype were determined at 38 weeks postinfection (Fig. 5I-5L and S7_Fig).

**Q4.** For the histology images in S5 and S6, it would be helpful to have the sections blindly scored to provide unbiased, quantitative data. By eye, some lung sections look different but are described in the results as being similar (especially in S5, 2 wpi), so more representative images or unbiased scoring would strengthen/clarify this data.

**Response:** We performed the requested new experiments including a quantitative lung inflammation score (S5E and S8E).

**Reviewer #2:**

**Q1**. I think the paper's conclusions regarding the role of neutrophils in the pathogenesis of MAI infection in this model are somewhat confusing. They note in Figure 3B that neutrophils have a "double peak" with increases early in infection and late, but that depletion of neutrophils in the first four weeks of infection does not influence mortality. However, it seems plausible that ongoing neutrophil depletion might provide some protection if prolonged. The authors conclude in line 319 that "neutrophils are not essential to the establishment of MAI infeciton." However, that's not really what this paper is about -- the high dose of MAI is important for the establishment of infection. I think the conclusions must be tempered.

**Response:** We modified the conclusions accordingly (page 16, paragraph 1, and line 341 – 348). We now treated infected mice with anti-Ly-6G monoclonal antibody after 32 weeks of infection and data is shown in Fig. 5I-5L and S7_Fig.

**Q2.** Models for NTM infection in small animals have not, to this point, been adequate surrogates for human disease. Furthermore, NTM disease is extremely diverse with skin, lung, bone, and disseminated infections and manifestations. It appears that this new model has characteristics of both fibronodular pulmonary infection (with increased lung disease) as well as disseminated infection (due to high bacterial burden systemically. I am curious how the authors feel that this model fits into this field -- What type of disease is this model most like?

**Response:** We think our model is primarily fibronodular pulmonary infection with a component of disseminated disease.

**Q3.** The rationale for choosing IL-17 as the treatment cytokine of choice based on data from Figure 3 and Figure S2 is not clear to me. Based on my reading of S2, it seems like many (32) cytokines were induced during the first 2 weeks of intranasal MAI infection. It might be useful to run a principal components analysis of all the cytokines in this data, then to define the groups of cytokines that are coexpressed with one another. I wouldn't be surprised some of the Th17 cytokines segregrated with one another. These might also provide clues toward cell specificity of cytokine responses when evaluated with flow cytometry cell analysis when available. Further, it will reduce the statistical burden of multiple comparisons in multiplexed cytokine data.

**Response:** As suggested, we now did principal components analysis (PCA). We noted that principal components (PC) 1 separated infected and uninfected mice efficiently (see below figure) but not in the infected group at different time points.

|  |  |
| --- | --- |
| **Principal components analysis (PCA)** | **Hierarchical clustering** |

Instead of PCA, we performed hierarchical clustering on cytokine and chemokine profiles obtained from 57 samples [uninfected mice (n = 15) and infected mice (n = 6 mice/each time point)]. Based on visual inspection of heat map and dendrogram, the expression profile of IL-17A is not similar to that of any other cytokines and chemokines tested in the multiplex ELISA (Fig 4A). Most of the cytokines and chemokines were elevated and reached peak after 2 - 3 weeks post-infection and gradually declined over time (Fig 4A and S2 Fig). On the contrary, IL-17A rapidly increased and reached peak at 1 – 2 weeks post-infection and then rapidly reduced after 3 weeks post-infection (Fig 4B). We selected IL-17A based on this unique expression profile.

**Reviewer #3:**

**Q1.** The examination of infection out to 39 weeks has revealed an unexpected imbalance in particular immune subsets, most strikingly a large accumulation of neutrophils at this late timepoint. Thus, it was surprising that there was only a focus on neutrophils on the establishment of infection, and the impact of neutrophil depletion at late stages of infection was not examined. This would be important to determine if this late accumulation of neutrophils is a key driver of pathology/mortality in this model.

**Response:** We have now performed these studies and the data is shown in Fig. 5I-5L and S7_Fig., and described on page 10, paragraph 2, and line 214 - 223.

**Minor Issues: Editorial and Data Presentation Modifications**

**Reviewer #1:**

**Q1**. If available, it would be nice to see images in Figure 1 or 2 of the whole lungs pre-sectioning to see if they had visible lesions like in lungs from Mtb-infected mice.

**Response:** We now showed the images (Fig 2E).

**Q2.** What was the endpoint used for time to death experiments? This should be included in the methods section.

**Response:** Mice were observed daily for signs of distress (ruffled fur, weight loss, reluctance to move, ataxia and labored respiration). For mortality studies, mice which lost 20% of their body weight were assessed by the Attending Veterinarian or Vivarium Staff to determine whether the mice required euthanization. Mortality was recorded until reached 50% - 75% of death. (Line 441 – 445 in the Materials and Methods section).

**Q3.** The time to death data indicates that some mice die over the course of infection—some early and more later. Did these deaths also occur in the infection done to characterize each individual time point? There’s no indication that there were deaths in these groups (all groups are equal in size at n=6), so I’m wondering if extra mice were available/infected to account for loses/deaths along the way? It might be worth addressing this minor discrepancy somewhere in the text.

**Response:** We performed a separate mortality study. Twenty mice per group were dedicated to the mortality study, and experiments were repeated two times. Thus, mortality was analyzed from a total of 40 mice. (Fig 1F and Line 134 – 142). Other experiments were performed with separate dedicated mice (Fig 1B-1E).

**Q4.** The panels from Fig 6G-H are duplicated in Fig S8.

**Response:** We now removed the duplicated supplementary figures.

**Q5.** I think labeling Fig 6C-M with the time point would help with clarity, especially because the schematic indicates multiple time points were assessed, but only one is presented in the main figure. Even though the info can be found in the legend, it’s nice to quickly see/understand that the data is from 39 weeks.

**Response:** We have now arranged these figures accordingly.

**Q6.** Line 253 mentions that MMP-9 levels are not affected by IL-17A blocking, but it doesn’t reference the MMP-9 data in Fig S9. The data in Fig S9E-F seem to show that MMP-9 does decrease—significantly so at 4 wpi—so a more accurate description might be that MMP-9 decreases transiently and modestly early in infection. I think this transient, modest decrease further stresses the importance of also examining MMP-9 levels in the in vitro infections in Figure 7.

**Response:** We revised these sentences accordingly (page 12, line 261 – 264). We also performed new in vitro experiments and results are shown in Fig. 7 and described on page 12 – 13, line 266 – 282.

**Q7.** The authors mention in the discussion the role of neutrophils during Toxoplasma and Listeria infection, but it would be useful to also mention the role of neutrophils during Mtb infection, especially since several other comparisons are drawn to Mtb throughout the discussion.

**Response:** We now mentioned the role of neutrophils in Mtb infection as requested (page 15, Line 334 – 337).

**Q8.** It would be interesting to know what aspects of human disease this model fails to recapitulate and maybe include in the discussion an analysis of what the limitations of this model are.

**Response:** We have now discussed these limitations as requested (page 14 - 15, paragraph 1 and Line 313 – 321).

**Q9.** In lines 329-330, the authors mention that the clinical strain used in this model is resistant to a couple antibiotics, but it’s not clear how that is related to IL-17A or early responses; what is the biological relevance/importance of this antibiotic resistance in this context?

**Response.** We removed the sentence to address this point.

**Reviewer #2: Minor issues:**

**Q1.** Line 136 or so: It is not clear to me the numbers of mice in each experimental group in Figure 1. It appears 40 total mice were used, but was this 1:1 segregation 2:1? 3:1? Please clarify

**Response:** As mentioned in response Reviewer #1 Q3 in Minor Issues, we performed a separate mortality study. Twenty mice per group were dedicated to the mortality study, and experiments were repeated twice. Thus, mortality was analyzed from a total of 40 mice. (Fig 1F and Line 134 – 137). Other experiments were performed with separate dedicated mice (Fig 1B-1E).

**Q2.** Figure 7. It doesn't appear that MAI induces significant MMP3 above baseline in airway epithelial cells in your in vitro system. However, MAI induces pulmonary destruction during MAI infection. Does this observation suggest the possibility of an alternate mechanism for tissue destruction in your model of MAI infection?

**Response:** As suggested by Reviewer #1 Q1 in Major Issues, we performed new *in vitro* experiments with different bacterial MOIs (0, 100 and 1000 MOI). MMP-3 levels were significantly increased with infection induced by 1000 MOI, not 100 MOI, of *M intracellulare*. This data indicates that a relatively high bacterial dose (1000 MOI) is required to induced MMP-3 production. Results are shown in Fig. 7 and described on page 12 – 13, line 266 – 282.

**Q3.** There are multiple grammatical errors throughout the manuscript and this paper would benefit from a scientific editor.

**Response:** All errors were corrected as suggested, we engaged a scientific editor, as suggested.

**Q4.** There was no description in the methods of how CT scanning and lung physiological function studies were performed in the mouse, thus no way to assess if this work was done appropriately. Please add details to how these studies were conducted, the equipment used, and necessary details for these experiments at all areas in the paper where they were used.

**Response:** We now described the imaging and physiologic studies in greater detail, as requested (page 24, line 519 – 535).

**Q5.** Figure 4D. I would like more details as to the total numbers of CD45CD3- and CD45CD3+ cells producing IL-17. Although CD3+ cells produce significant IL-17, I would not be surprised if another cell type might have a greater frequency of cells that produce IL-17 (ILC3, for example). It seems a bit of a stretch to state that CD3+ cells are the primary cell type producing IL-17 when the flow panel is as sparse as it is.

**Response:** We now performed additional new experiments to address this point and the data is now shown in Fig. 4C and S3.

**Reviewer #3:**

**Q2.** In relation to the point above, the authors may wish to expand upon discussion of the potential role of KC during chronic infection, considering its association with neutrophil accumulation and heightened levels at 39 weeks post-infection.

**Response:** KC is a powerful chemoattractant that recruits neutrophils to sites of infection. In the present study, KC was re-increased in the lung of mice during chronic infection. It suggests that KC might be association with neutrophil accumulation during chronic infection. However, depletion of neutrophils during chronic infection had no effect on mortality, lung damages and bacteria growth in chronically infected mice (Fig. 5I-5L and S7_Fig.). Therefore, we do not discuss about the potential role of KC in the current study.

**Q3.** The study required a very large dose of bacteria to achieve bacterial persistence and eventual mortality; the starting CFU seeded to the lung is >10e7 bacteria, and numbers increase by just over 10-fold after 39 weeks. Thus it is not necessarily true that this model ‘closely resembles the clinical presentation of human chronic NTM infection’ (see line 295); indeed the mortality incurred here could be to the unusually high dose delivered intranasally, which results in a much greater seeding of the lung compared to aerosol delivery (see reference 34 where aerosol delivery of a lower dose of bacteria resulted in greater initial replication and subsequent bacterial persistence). The authors may wish to better discuss the limitations of their model, considering the points raised above.

**Response:** We concur and have revised these sentences. We also discuss the limitations of our model (page 14 - 15, paragraph 1, and line 313 – 321).

**Q4.** Interpretation of some of the data needs to be improved. The abstract (line 30) states that ‘depletion of neutrophils during the early 2 weeks of infection had no effect on bacterial burden, structure and function of lung and mortality until 48 weeks of infection’, however the data shows no difference at this late stage. Similarly, Line 368 states that ‘early IL-17A production helps M. intracellulare to establish infection in mouse lungs’, but the data shows that the impact of IL-17 depletion is most apparent at later stages of infection.

**Response:** We revised the abstract (page 2, line 31 – 34). IL-17A was increased in the lungs during early infection (1–2 weeks) and neutralization of IL-17A during this period of infection (0 – 2 weeks) significantly reduced bacterial burden, fibrotic lung damage, and mortality in chronically infected mice (at 39 weeks postinfection). This imply that IL-17A is required to establish infection (early infection) and subsequently other factor(s) are required to progression of disease. Future studies should be conducted on the disease stage of interest.

**Q5.** Bar graphs should show individual data points for each animal.

**Response:** As suggested, we now show individual data points for each animal.

**Q6.** The study would benefit from a more through proof read to fix grammatical errors.

**Response:** We acknowledge the point and have engaged a scientific writer to help improve the presentation and have proof-read the text to correct grammatical errors.
